# Synthetic Fragments of Receptor for Advanced Glycation End Products Bind Beta-Amyloid 1–40 and Protect Primary Brain Cells From Beta-Amyloid Toxicity

**DOI:** 10.3389/fnins.2018.00681

**Published:** 2018-09-27

**Authors:** Anna V. Kamynina, Noemi Esteras, Dmitriy O. Koroev, Natalia V. Bobkova, Samson M. Balasanyants, Ruben A. Simonyan, Armine V. Avetisyan, Andrey Y. Abramov, Olga M. Volpina

**Affiliations:** ^1^Shemyakin-Ovchinnikov Institute of Bioorganic Chemistry, Russian Academy of Sciences, Moscow, Russia; ^2^Department of Clinical and Movement Neurosciences, Institute of Neurology, University College London, London, United Kingdom; ^3^Institute of Cell Biophysics, Russian Academy of Sciences, Pushchino, Russia; ^4^Belozersky Institute of Physico-Chemical Biology, Moscow State University, Moscow, Russia

**Keywords:** beta-amyloid, synthetic peptides, receptor for advanced glycation end products, Alzheimer’s disease, primary cell culture

## Abstract

Receptor for advanced glycation end products (RAGE) is involved in the pathogenesis of Alzheimer’s disease. We have previously revealed that RAGE fragment sequence (60–76) and its shortened analogs sequence (60–70) and (60–65) under intranasal insertion were able to restore memory and improve morphological and biochemical state of neurons in the brain of bulbectomized mice developing major AD features. In the current study, we have investigated the ability of RAGE peptide (60–76) and five shortened analogs to bind beta-amyloid (Aβ) 1–40 in an fluorescent titration test and show that all the RAGE fragments apart from one [sequence (65–76)] were able to bind Aβ *in vitro*. Moreover, we show that all RAGE fragments apart from the shortest one (60–62), were able to protect neuronal primary cultures from amyloid toxicity, by preventing the caspase 3 activation induced by Aβ 1–42. We have compared the data obtained in the present research with the previously published data in the animal model of AD, and offer a probable mechanism of neuroprotection of the RAGE peptide.

## Introduction

The receptor for advanced glycation end products (RAGE) is a multiligand polyfunctional receptor, which takes part in the pathology of many diseases (Deane, 2012). In particular, RAGE is involved in the pathology of Alzheimer’s disease (AD) by activating neuroinflammation (Ramasamy et al., 2009), inducing neuronal dysfunction (Perrone et al., 2012), and mediating the transport of pathological beta-amyloid (Aβ) from the blood into the brain (Yan et al., 2010). It has been shown that in human AD brains, the level of RAGE expression is increased in neuronal and microglial cells and its expression is correlated to the severity of the disease (Yan et al., 1996; Lue et al., 2001; Leclerc et al., 2010). Transgenic mice overexpressing RAGE in neurons or microglia demonstrated enhanced Aβ production, enhanced neurotoxicity, reduced cognition and enhanced inflammation (Walker et al., 2015).

Previously, we had shown that synthetic RAGE fragment sequence (60–76) protects spatial memory of mice with an experimentally induced form of AD (olfactory bulbectomized mice),improves the morphological and functional state of the neurons and lowers the level of brain Aβ in experimental animals (Volpina et al., 2015, 2018). In our research, we revealed that shortened RAGE fragments have also a protective activity in the animal model: fragment sequence (60–70) has almost the same activity as (60–76), while fragment (60–65) was less effective (Volpina et al., 2018). These results obtained in the animal model led us to investigate in more detail the mechanism of neuroprotection of the RAGE peptide (60–76).

We demonstrated that peptide (60–76) binds amyloid plaques in Tg-5xFAD mice brain slices and Aβ 1–40 trimer in low SDS electrophoresis. We proposed that one of the possible protective pathways of the peptide (60–76) might be the result of its binding to Aβ. To identify the conditions under which the RAGE fragment develops its protective activity, we have investigated whether peptide (60–76) and its shortened analogs bind Aβ *in vitro*.

One of the key stages in AD development is the death of neurons and astrocytes in the hippocampus and cortex (Sadigh-Eteghad et al., 2015; Hardy and Selkoe, 2016). In order to reveal the protective activity of the chosen RAGE fragments *in vitro*, in the current research we have also investigated whether the RAGE peptides were able to reduce the amyloid-induced toxicity in Aβ 1–42-treated primary cultures of neurons and astrocytes derived from hippocampal and cortical areas of rats’ brains.

The data obtained in the present study have been compared with our previous results showing the protective activity of the RAGE fragments in the animal model of AD (olfactory bulbectomized mice), and we have identified the most active RAGE fragment. Here, we also discuss the possible mechanism of neuroprotection of the active peptide.

## Materials and Methods

### Peptide Synthesis

RAGE fragments presented in **Table 1** were derived from the sequences of human RAGE (Q15109 UniProtKB/SwissProt). Human Aβ1–40 was derived from sequences of human amyloid precursor protein (P05067.3, UniProtKB/Swiss-Prot). RAGE peptides and Aβ1–40 were synthesized on the Wang resin using the Fmoc/Bu^t^-scheme as described previously in Volpina et al. (2018). The peptides were purified by HPLC on C18 column (Phenomenex Jupiter 10 μ C18 300A 250 × 10 mm) in the acetonitrile gradient from 10 to 70% in 0.1% TFA (registration at 226 nm). Synthetic peptides were characterized by analytical reversed-phase HPLC on C18 column (Phenomenex Jupiter 5 μ C18 300A 250 × 4.6 mm) and MALDI-MS on VISION 2000 (Bioanalysis, United Kingdom). Purity of the peptides was estimated as > 95%.

**Table 1:** Amino acid sequences of the chosen RAGE fragments.

Abbreviation	Number	A.a. sequence
P1	60–76	AWKVLSPQGGGPWDSVA
P2	60–70	AWKVLSPQGGG
P3	60–62	AWK
P4	60–65	AWKVLS
P5	65–76	SPQGGGPWDSVA
P6	62–73	KVLSPQGGGPWD


### Fluorescent Titration of RAGE Fragments by Aβ 1–40

The fluorescence titrations of synthetic peptides were performed using Fluoromax 3 fluorescence spectrophotometer (Horiba Jobin Yvon, Germany). Titrations were conducted in a quartz cuvette with 2 ml of titration buffer (25 mM Tris-HCl, 100 mM NaCl, 5 mM EDTA, pH 7.5). The concentration of peptide (60–76) which has two tryptophan residues was 0.5 μM. Whereas peptides (60–70), (60–62), (60–65), (62–73), and (65–76) have only one Trp residue, their concentration was 2 μM to enhance the signal. Lyophilized synthetic Aβ 1–40 was dissolved in mili-Q water at a concentration of 1 mM. In experiments with (60–76), beta-amyloid was diluted in titration buffer to 100μM and added with the step of 100 nM to form concentrations from 100to 1500 nM in the cuvette. During the titrations of (60–70), (60–62), (60–65), (62–73), and (65–76) we added increasing concentrations of Aβ 1–40 at 200, 400, 800, 1200, 1600, 2000, 2400, 2800, 3200, 3600, and 4000 nM, which was previously diluted 2.5 times.

The fluorescence spectrum was measured after each addition of beta-amyloid. The excitation wavelength was λ = 285 nm. Emission was detected in the range of 250–450 nm. The maximum of fluorescence intensity was found at 355 nm. In Statistica 10 (StatSoft United States) the set of intensities were distributed on a plot with coordinates 1/[S] versus 1/ΔF and were fitted in the linear equation:

$$\frac{1}{\Delta F}=\frac{1}{\Delta F_{\max}}+\frac{K_{d}}{\Delta F_{\max}}\frac{1}{\left[S\right]}$$

where [S] – concentration of Aβ 1–40 in cuvette; ΔF is the difference of intensity between experiment in absence of Aβ 1–40 and experiment with beta-amyloid at the certain concentration and ΔFmax is the maximal fluorescence change (Huang et al., 2016). The linear distribution was plotted by least square method and characterized by the coefficient of determination *R*^2^ and standard error of estimation (SEE). These values are presented at **Table 2**. The distribution was acceptable when *R*^2^ ≥ 0.8.

**Table 2:** Characteristics of linear distributions obtained during the fluorescent titrations of peptides P1–P6.

Peptide	*R*^2^	SEE
P1	0.9799	0.000008503
P2	0.9975	0.000000678
P3	0.9931	0.000000236
P4	0.9336	0.000072342
P5	0.0443	0.000250921
P6	0.9678	0.000005964


*R^2^ for all peptides, except P5, are more than R^2^ = 0.8.*

The dissociation constant K_d_ was found by multiplication of the calculated slope factor and ΔFmax. For each peptide the dissociation constant was measured three times independently. Dissociation constants were expressed as mean ± SEM. *P*-values were measured by Mann-Whitney test to estimate the differences between each of obtained dissociation constants.

### Cell Culture

Mixed cultures of hippocampal and cortical neurons and glial cells were prepared as described previously (Vaarmann et al., 2010) with modifications, from Sprague-Dawley rat pups 2–4 days post-partum (UCL breeding colony). Experimental procedures were performed in full compliance with the United Kingdom Animal (Scientific Procedures) Act of 1986 and with approval of the University College London Animal Ethics Committee. Hippocampi and cortex were removed into ice-cold PBS (Ca^2+^, Mg^2+^-free, Invitrogen, Paisley, United Kingdom). The tissue was minced and trypsinised (0.25% for 15 min at 37°C), triturated and plated on Poly-D-lysine-coated coverslips and cultured in Neurobasal A medium (Invitrogen, Paisley, United Kingdom) supplemented with B-27 (Invitrogen, Paisley, United Kingdom) and 2 mM L-glutamine. Cultures were maintained at 37°C in a humidified atmosphere of 5% CO_2_ and 95% air, fed twice a week and maintained for a minimum of 12 days before experimental use to ensure expression of glutamate and other receptors. Neurons were easily distinguishable from glia: they appeared phase bright, had smooth rounded somata and distinct processes, and lay just above the focal plane of the glial layer. Cells were used at 12–15 days *in vivo* (DIV) unless otherwise stated.

### Caspase 3 Activity Assay

For the measurement of caspase 3 activation, cells were loaded for 15 min at room temperature with 10 μM NucView 488 caspase 3 substrate (Biotium, United States) in HBSS. NucView 488 is a novel class of enzyme substrates for real-time detection of caspase-3 activity in live cells. The substrate can rapidly cross cell membrane to enter the cell cytoplasm, where it is cleaved by caspase-3 to release the high-affinity DNA dye. The released DNA dye migrates to the cell nucleus to stain the nucleus brightly green. Cells were pre-incubated for 3 h with 10 μM of each peptide. Then, cells were treated for 1 h with 5 μM Aβ 1–42 (Bachem, Cambridge Bioscience). Beta-amyloid 1–42 was prepared as described previously in Narayan et al. (2014). Confocal images were obtained using Zeiss (Oberkochen, Germany) 710 confocal laser scanning microscope and a 40× oil immersion objective. The 488 nm argon laser was used to excite NucView 488 fluorescence, which was measured using a bandpass filter from 510 and 560 nm. All of the data shown were obtained from at least 5 coverslips and 2–3 different cell preparations. N corresponds to the number of fields taken for cell calculation from coverslips. The number of cells with activated caspase 3 and the number of all visible cells was quantified manually in each field and the percentage of dead cells was calculated by dividing the number of caspase 3-positive cells by the number of all cells counted in one field. The differences between the groups were evaluated using the two-sample *t*-test. All data were expressed as mean ± SEM. The results in the figure are presented as mean ± SD.

## Results

### Synthetic Peptides

In this study we used a panel of synthetic peptides listed in **Table 1** including full-size peptide (60–76) and truncated overlapping fragments. Peptides (60–70), (60–65), and (60–62) are truncated at the C-terminus fragments of the peptide (60–76). The peptide (62–73) was shortened both to the N-terminal and to the C-terminal sequence. The peptide (65–76) is devoid of the N-terminal sequence.

### Fluorescent Titration of RAGE Fragments by Aβ 1–40

**FIGURE 1:**
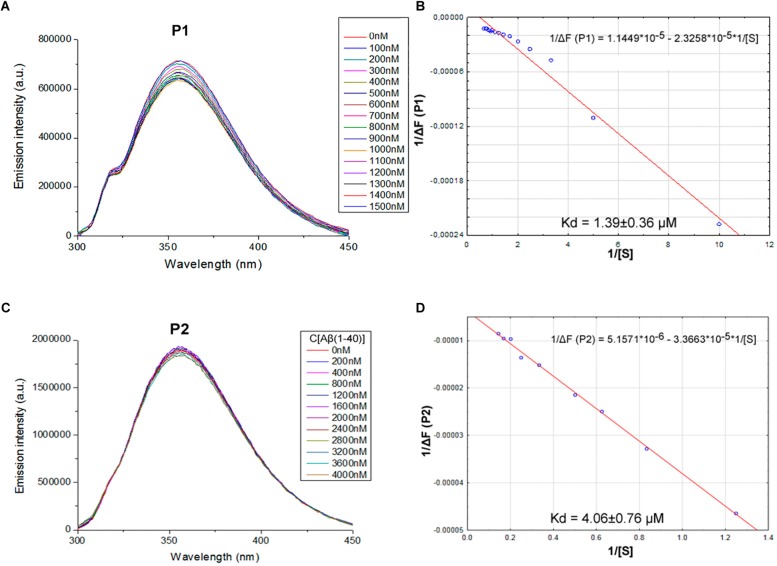
Identification of the binding affinities of peptides P1 **(A,B)** and P2 **(C,D)** with Aβ 1–40 by fluorescence spectroscopy. **(A)** Emission spectra of 0.5 μM P1 peptide fluorescence titrations exhibit decreasing fluorescence intensities with increasing concentration of Aβ 1–40 in range 0–1500 nM. **(B)** Curve with P1 fluorescence intensity changes versus Aβ 1–40 concentrations measured at a wavelength of 355 nm. **(C)** Emission spectra of 2 μM P2 peptide fluorescence titrations exhibit decreasing fluorescence intensities with increasing concentration of Aβ 1–40 in range 0–4000 nM. **(D)** Curve with P2 fluorescence intensity changes versus Aβ 1–40 concentrations measured at a wavelength of 355 nm. Linear equations were utilized for the dissociation constants (K_d_) calculations **(B,D)**.

**FIGURE 2:**
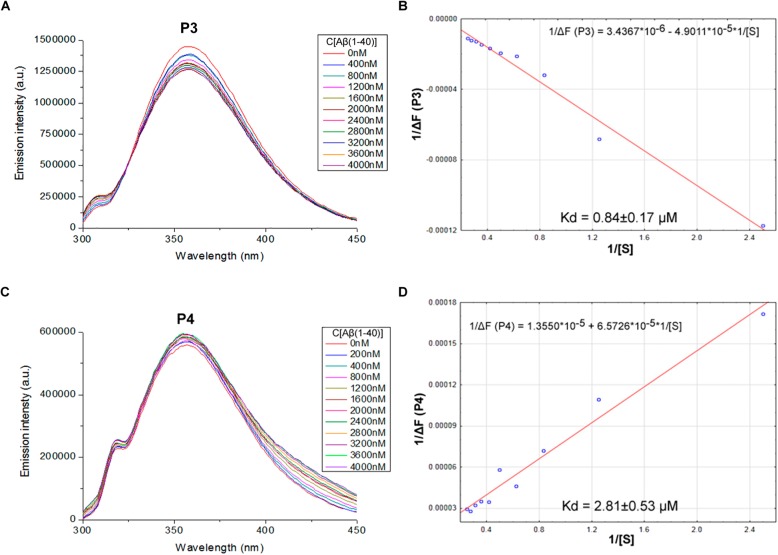
Identification of the binding affinities of peptides P3 **(A,B)** and P4 **(C,D)** with Aβ 1–40 by fluorescence spectroscopy. **(A)** Emission spectra of 2 μM P3 peptide fluorescence titrations exhibit decreasing fluorescence intensities with increasing concentration of Aβ 1–40 in range 0–4000 nM. **(B)** Curve with P3 fluorescence intensity changes versus Aβ 1–40 concentrations by measurement at a wavelength of 355 nm. **(C)** Emission spectra of 2 μM P4 peptide fluorescence titrations exhibit increasing fluorescence intensities with increasing concentration of Aβ 1–40 in range 0–4000 nM. **(D)** Curve with P4 fluorescence intensity changes versus Aβ 1–40 concentrations measured at a wavelength of 355 nm. Linear equations were utilized for the dissociation constants (K_d_) calculations **(B,D)**.

**FIGURE 3:**
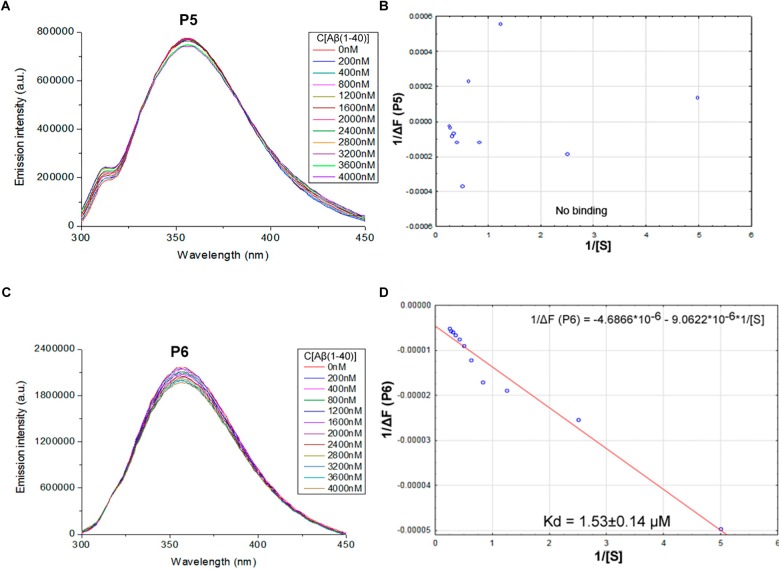
Identification of the binding affinities of peptides P5 **(A,B)** and P6 **(C,D)** with Aβ 1–40 by using fluorescence spectroscopy. **(A)** Emission spectra of 2 μM P5 peptide fluorescence titrations exhibit the absence of consecutive changes in fluorescence intensities with increasing concentration of Aβ 1–40 in range 0–4000 nM. **(B)** Curve with P5 fluorescence intensity changes versus Aβ 1–40 concentrations by measurement at a wavelength of 355 nm. The non-linear fitting of P5 titration data demonstrated the absence of binding with Aβ 1–40 and did not allow finding a dissociation constant. **(C)** Emission spectra of 2 μM P6 peptide fluorescence titrations exhibit decreasing fluorescence intensities with increasing concentration of Aβ 1–40 in range 0–4000 nM. **(D)** Curve with P6 fluorescence intensity changes versus Aβ 1–40 concentrations measured at a wavelength of 355 nm. Linear equation was utilized for the dissociation constant (K_d_) calculations **(D)**.

We proposed previously that one of the protection pathways influenced by the peptide (60–76) is the interaction of P1 with Aβ. Fluorescent titrations of P1 peptide and its fragments by Aβ 1–40 were conducted to study their ability to bind beta-amyloid *in vitro*. We preferred this method because it did not require using any additional fluorescent label, but only the peptide tryptophan residues as the source of fluorescence, which preserves the native peptide conformation and solubility. It is known that tryptophan fluorescence is very sensitive to the binding of the target peptides with ligands (Huang et al., 2016). Aβ 1–40 was utilized for an accurate control of the concentrations in the cuvette due to its higher solubility in comparison with the Aβ 1–42 isoform (Olivero et al., 2014). During the titration of P1–P6 fragments, the intensity of emission changes linearly for five fragments – for P1, P2, P3, P4, and P6 (**Figures 1**–**3**).

The data of P5 titration was irregular and did not give a strong linear distribution (*R*^2^ < 0.8). It was shown that peptides P1–P4 and P6, but not the fragment truncated at the N-terminus (P5) (**Figure 3**), specifically bound to Aβ 1–40. The dissociation constants for complexes of these peptides with Aβ 1–40 are presented in the **Table 3**. The dissociation constant (K_d_) for full-size P1 peptide was established at the level of ∼1.4 μM. The values of K_d_ for P3, P4, and P6 fragments were found equal with the K_d_ for P1 (*p* > 0.05). The only shortened peptide with a significantly different dissociation constant to P1 (*p* = 0.045) was P2 with a K_d_ of around 4.1 μM. To sum up, the fluorescence titrations demonstrated that the interactions between Aβ 1–40 and all of the peptides, except P5, are at the micromolar level, provided that the amyloid-peptide complexes are very stable in the conditions of this experiment (Huang et al., 2016).

**Table 3:** Interactions between RAGE fragments and Aβ 1–40.

Peptide	K_d_, μM^∗^	*p*-value^∗∗^
P1	1.39 ± 0.36	
P2	4.06 ± 0.76	0.045
P3	0.84 ± 0.17	0.716
P4	2.81 ± 0.53	0.112
P5	No binding	–
P6	1.53 ± 0.14	0.377


*^∗^The dissociation constants K_*d*_ are presented as mean ± SEM of three independent experiments. ^∗∗^p-values were calculated to estimate the statistical significance between the dissociation constants for P2–P6 fragments versus K_*d*_ for P1 peptide.*

### Caspase-3 Activation Test

To investigate the protective activity of the RAGE peptides, we treated primary cultures of neurons and astrocytes derived from hippocampal and cortical areas of rats’ brains, with Aβ 1–42. Aβ can trigger the cell death cascade by activation of caspase 3 (Harada and Sugimoto, 1999; Kamynina et al., 2013). For the evaluation of the protective activity of the RAGE fragments, the culture was preliminary incubated for 3 h with each of the six peptides. After that, the culture was incubated for 1 h with Aβ 1–42. The visualization of the caspase 3 activation in real time was conducted using NucView 488 caspase 3 substrate.

**FIGURE 4:**
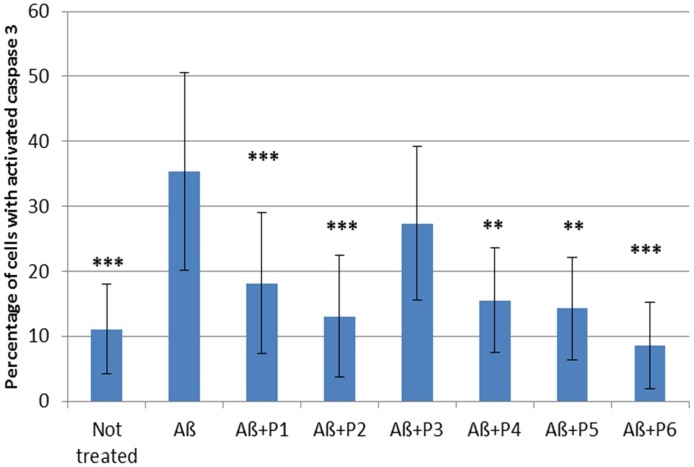
Percentage of cells with activated caspase 3 following application of Aβ 1–42 and one of the RAGE fragments to primary cultures of neurons and astrocytes derived from hippocampal and cortical areas of rats’ brains (*N* = 6–28 fields). Percentage of dead cells was calculated by dividing the number of cells with activated caspase 3 (cells with green nuclei) by the number of all cells counted in one field. The data are presented as mean ± SD. ^∗^*p* < 0.05; ^∗∗^*p* < 0.01; ^∗∗∗^*p* < 0.001; Aβ 1–42 versus not treated culture; or Aβ 1–42 versus RAGE fragments (P1–P6).

**FIGURE 5:**
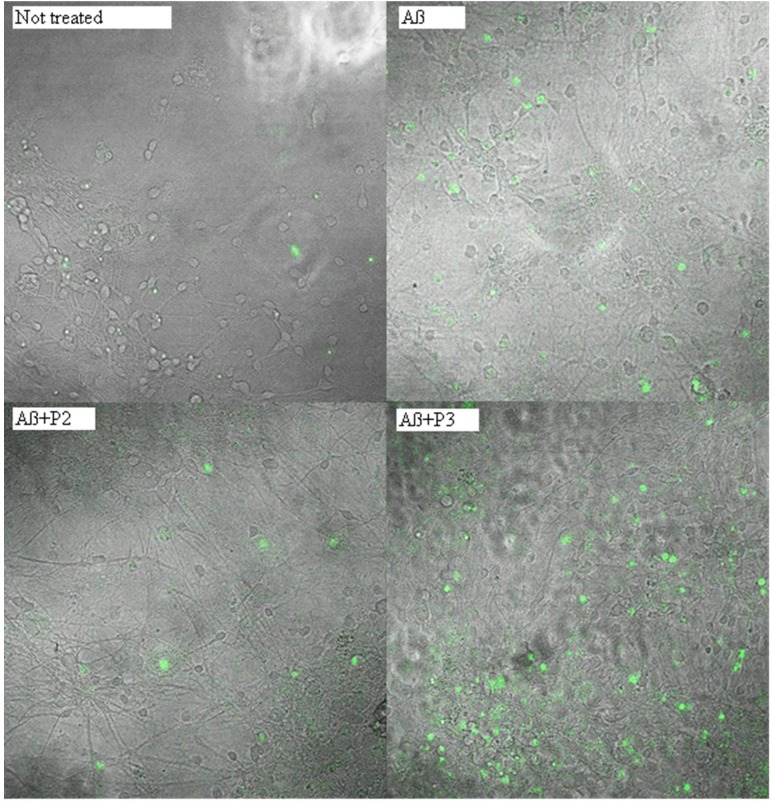
Photomicrographs of cells with activated caspase 3 after treatment with beta-amyloid 1–42 and peptides P2 or P3. Green nuclei appear in the cells with activated caspase 3.

It was shown that application of Aβ 1–42 induced a rapid activation of caspase 3 in neurons and astrocytes derived from hippocampal and cortical areas of rats’ brains (percentage of cells with activated caspase 3 = 35.345 ± 3.41; *N* = 20, **Table 4** and **Figure 5**) in comparison with the control non-treated culture (percentage of dead cells = 11.075 ± 1.54; *N* = 20). It should be noted that pre-incubation of the cultures with five RAGE fragments (P1, P2, P4, P5, and P6) reduced the Aβ 1–42 induced rate of appearance of caspase 3 activation and the percentage of dead cells with green nuclei with different significance (*N* = 6–28) (**Table 4** and **Figures 4**, **5**). The most protective activity was achieved with fragments P1, P2, and P6, which were able to significantly reduce Aβ activated caspase 3 (*p* < 0.001, percentage of dead cells = 18.13 ± 2.05; *N* = 28, 13.07 ± 2.71; *N* = 12 and 8.53 ± 2.72; *N* = 6, respectively). The activity of Aβ-activated caspase 3 was also significantly (*p* < 0.01) reduced after pre-incubation with P4 (percentage of dead cells = 15.5 ± 3.27; *N* = 6) and P5 (percentage of dead cells = 14.25 ± 3.22; *N* = 6). Only peptide P3 did not decrease the rate of Aβ-induced caspase 3 activation (number of cells = 27.37 ± 2.79; *N* = 18, **Figure 5**).

**Table 4:** Percentage of cells with activated caspase 3 following application of Aβ 1–42 and one of the RAGE fragments to primary cultures of neurons and astrocytes derived from hippocampal and cortical areas of rats’ brains.

	N	Mean, %	SEM	*p*-value^a^	
Not treated	20	11.075	1.53647	<0.0001	^∗∗∗^
Aß 1–42	20	35.345	3.41302		
Aß + P1	28	18.12857	2.04813	<0.0001	^∗∗∗^
Aß + P2	12	13.06667	2.70771	<0.0001	^∗∗∗^
Aß + P3	18	27.36667	2.79309	0.0827	–
Aß + P4	6	15.53333	3.26748	0.00583	^∗∗^
Aß + P5	6	14.25	3.22229	0.00361	^∗∗^
Aß + P6	6	8.53333	2.72013	<0.0001	^∗∗∗^


*^∗^*p* < 0.05; ^∗∗^*p* < 0.01; ^∗∗∗^*p* < 0.001.*

*N corresponds to the number of fields. The number of cells with activated caspase 3 and the number of all visible cells was quantified manually in each field and the percentage of dead cells was calculated by dividing the number of dead cells by the number of all cells counted in one field.*

*^*a*^p-values were calculated to estimate statistical significance between the group of cells after application of Aβ 1–42 versus group of not treated culture or the culture treated with Aβ 1–42 and one of the RAGE fragments (P1–P6).*

## Discussion

Previously, we have shown that intranasal administration of RAGE fragments resulted in the improvement of both spatial memory and morpho-functional characteristics in the brain of mice with experimentally induced AD (Volpina et al., 2018). Peptide P1 (60–76) showed the most successful activity in both parameters. Administration of the shorter fragment P2 (60–70) also prevented memory loss in the experimental animals. The short peptide P4 (60–65) had protective activity on mouse memory, but not significantly different from the control group of non-treated mice. Experiments in the animal model also showed the binding of peptide (60–76) with amyloid plaques. This suggested that active fragments may exert their protective action through their binding with Aβ (Volpina et al., 2018). To test this hypothesis we investigated the binding of the synthetic fragments with Aβ 1–40 by fluorescent titration. Aβ 1–40 is known to have more solubility (Olivero et al., 2014; Iljina et al., 2016), than Aβ 1–42, which makes this isoform more preferable for *in vitro* binding experiments. As a result of the fluorescent titration, we have depicted that five of the fragments (P1, P2, P3, P4, and P6) were able to bind Aβ 1–40 *in vitro* with micromolar level of K_d_. Only one fragment, P5, did not form a complex with Aβ under these conditions.

To investigate the effect of the RAGE fragments on Aβ toxicity, we evaluated the rate of caspase 3 activation in primary neuronal cultures, after the application of Aβ and the RAGE fragments. Since Aβ 1–42 is known to have more toxic effects in comparison with Aβ 1–40 (Favaloro et al., 2012; Pauwels et al., 2012; Cieślik et al., 2015), for the caspase 3 assays we used Aβ peptide 1–42. In the present research, we show that treatment of the culture with Aβ 1–42 results in a significant enhancement of caspase 3 activation and hence, cell death. Pre-incubation of the culture with P1 (60–76) and its shortened analogs – P2 (60–70), P4 (60–65), P5 (65–76) and P6 (62–73), leads to significant protection of the cells from Aβ-induced death. It should be noted that P1 (60–76), P2 (60–70), and P6 (62–73) fragments demonstrated the most protective activity on the rate of caspase 3 induction.

We have also compared the data obtained in the present study with the previously obtained data demonstrating the protective activity of the peptides on the memory loss of experimental animals (**Table 5**). The most active fragments in the animal model, peptides P1 (60–76) and P2 (60–70), were able not only to significantly prevent the activation of cell death in response to addition of Aβ, but also bind Aβ *in vitro*.

**Table 5:** Effects of the RAGE peptides observed in animal tests, caspase 3 activity, study and fluorescent titration by Aβ 1–40.

Peptide	Number	Memory protection (Volpina et al., 2018)	Caspase 3 activity	Aβ 1–40 binding
P1	60–76	+	+	+
P2	60–70	+	+	+
P3	60–62	–	–	+
P4	60–65	±	+	+
P5	65–76	–	+	–
P6	62–73	–	+	+


*“+,” indicates the presence of positive effect; “-,” its absence.*

The short peptide P4 (60–65), showed protective activity in the animal test, but not significantly different from the activity of the peptide P2 (60–70), and the statistical processing of the obtained data did not allow to obtain reliable differences from the group of not treated bulbectomized animals. For this reason, the data for this peptide in **Table 5** are shown as ±. As a result of the present research it was revealed that peptide P4 was able to protect cells from Aβ induced cell death and bind Aβ.

Peptides P3, P5, and P6 did not prevent memory loss occurring in the experimental mice as shown in our previous study. However, these fragments act differently in the present research. In particular, fragment P3 did not inhibit Aβ toxicity in cells, but bound Aβ 1–40; fragment P5 did not bind Aβ 1–40, but significantly decreased the number of cells with activated caspase 3 appearing in response to the application of Aβ 1–42. Peptide P6 induced both effects – it made a complex with Aβ and prevented amyloid toxicity in the primary culture.

Thus, it seems that for the manifestation of a protective activity in the animal tests, peptides should both inhibit caspase 3 activation induced by toxic Aβ and bind Aβ *in vitro*. Considering the fact that some of the RAGE peptides which protected cells from beta-amyloid toxicity did not bind beta-amyloid in the fluorescent titration test (for instance, P5), we propose that their protective activity is not connected only with binding with amyloid but they are more likely to mediate physiological functionality of the RAGE receptor. The mechanism of memory protection appeared to be more complicated than we had believed earlier. From one hand, binding with Aβ and prevention of caspase activity offers only one of the possible explanations why peptides preserve memory state of olfactory bulbectomized animals. At the same time, manifestation of both of these effects for a peptide is not sufficient to stop memory loss progression observed in the experimental animals. The mechanism of the protective activity of the peptides is likely to be a result of additional pathways which are expected to be revealed.

Despite numerous investigations, the role of RAGE molecule in the pathology of AD is still not clear enough. The failure of the third stage of clinical trials of azeliragon, which blocks the binding of RAGE with its ligands (Godyn et al., 2016), has demonstrated that participation of RAGE in AD is more complicated than just binding with beta-amyloid. The data shown here, regarding the activity of RAGE peptide (60–76) gives new opportunities toward further investigation of the role of this receptor in AD pathology and provides new pathways for a potential drug treatment of this disease.

## Author Contributions

AK prepared primary cell culture, conducted amyloid treatment and peptide application, made caspase 3 activity test, and prepared drafted manuscript. SB made fluorescent titration of RAGE fragments by Aβ 1–40 and prepared figures for the drafted manuscript. DK made peptide synthesis and characterization of peptides. NE made statistical analysis of caspase 3 activity test. RS made data counting of fluorescent titration and dissociation constant measurement. AVA made statistical analysis of fluorescent titration. NB conducted the purification of peptides. AYA provided interpretation of the data and confocal analysis of NucView caspase 3 test. OV was responsible for the conception of the work and contributed to the discussion of the manuscript.

## Conflict of Interest Statement

The authors declare that the research was conducted in the absence of any commercial or financial relationships that could be construed as a potential conflict of interest. The reviewer MB and handling Editor declared their shared affiliation.
